# Supplementary material for: Resolving the full spectrum of human genome variation using Linked-Reads
Source: Genome Res. 2019 Apr;29(4):635–45. doi: 10.1101/gr.234443.118 (PMC6442396; doi:10.1101/gr.234443.118)
Supplement: Supplemental Material [file supp_gr.234443.118_Supplemental_longranger-2.2.zip › longranger-2.2.tar.bz2!/lr2.2-public/lib/stan/cmdstan/stan/doc/api/html/no__op_8hpp_source.html]

Stan: src/stan/common/recorder/no\_op.hpp Source File


|  |  |
| --- | --- |
|  | Stan  2.5.0  probability, sampling & optimization |


- Main Page
- Related Pages
- Namespaces
- Classes
- Files

- File List
- File Members

All Classes Namespaces Files Functions Variables Typedefs Enumerations Enumerator Friends Macros Pages

- src
- stan
- common
- recorder

no\_op.hpp

Go to the documentation of this file.

1 #ifndef STAN\_\_COMMON\_\_RECORDER\_\_NO\_OP\_HPP

2 #define STAN\_\_COMMON\_\_RECORDER\_\_NO\_OP\_HPP

3

4 #include <ostream>

5 #include <string>

6 #include <vector>

7

8 namespace stan {

9  namespace common {

10  namespace recorder {

11

15  class no\_op {

16  public:

23  template <class T>

24  void operator()(const std::vector<T>& x) { }

25

31  void operator()(const std::string x) { }

32

37  void operator()() { }

38

42  bool is\_recording() const {

43  return false;

44  }

45  };

46

47

48  }

49  }

50 }

51

52 #endif

stan::common::recorder::no\_op

Does nothing.

**Definition:** no\_op.hpp:15

stan::common::recorder::no\_op::operator()

void operator()(const std::string x)

Do nothing with a string.

**Definition:** no\_op.hpp:31

stan::common::recorder::no\_op::operator()

void operator()()

Do nothing.

**Definition:** no\_op.hpp:37

stan::common::recorder::no\_op::is\_recording

bool is\_recording() const

Indicator function for whether the instance is recording.

**Definition:** no\_op.hpp:42

stan::common::recorder::no\_op::operator()

void operator()(const std::vector< T > &x)

Do nothing with vector.

**Definition:** no\_op.hpp:24


---

[ Stan Home Page ]

*© 2011–2014,
Stan Development Team.*
